# Dietary Flavonoids Vitexin and Isovitexin: New Insights into Their Functional Roles in Human Health and Disease Prevention

**DOI:** 10.3390/ijms26146997

**Published:** 2025-07-21

**Authors:** Weiqi Yan, Junying Cheng, Baojun Xu

**Affiliations:** 1 Food Science and Technology Program, Department of Life Sciences, Beijing Normal-Hong Kong Baptist University, Zhuhai 519087, China; yanweiqi@uic.edu.cn (W.Y.); r130013004@mail.uic.edu.cn (J.C.); 2 Centre for Cancer and Inflammation Research, School of Chinese Medicine, Hong Kong Baptist University, Hong Kong SAR, China

**Keywords:** vitexin, isovitexin, dietary flavonoid, chronic disease prevention, bioavailability, health promotion

## Abstract

Vitexin and isovitexin are dietary flavonoids widely distributed in food and medicinal plants. They have attracted increasing attention owing to their diverse pharmacological activities and favorable safety profiles. These compounds exhibit therapeutic potential across multiple biological systems, including the immune, nervous, respiratory, cardiovascular, and endocrine systems, through antioxidant, anti-inflammatory, anticancer, antibacterial, and neuroprotective mechanisms. Although previous reviews have addressed the pharmacological effects of vitexin and isovitexin, most are limited in scope—either focusing solely on vitexin or restricted to specific disease models such as cancer or diabetes. Moreover, some studies are outdated and do not reflect the recent advances in synthetic modification, green extraction technologies, and systems pharmacology. This review aims to provide a comprehensive evaluation of the pharmacological properties, pharmacokinetics, and clinical relevance of vitexin and isovitexin, highlighting their potential in disease prevention and treatment. A literature search was conducted using Web of Science, PubMed, and Google Scholar, with keywords including “vitexin”, “isovitexin”, “disease”, and “mechanism”. Here, we summarize the current research on the pharmacological effects of vitexin and isovitexin in metabolic disorders, inflammatory diseases, cancer, and neurodegenerative conditions, focusing on their molecular mechanisms and therapeutic targets. Furthermore, we discussed their toxicity, bioavailability, pharmacokinetics, and clinical research findings. Vitexin and isovitexin hold promise as therapeutic agents or adjuncts for multiple diseases with potential applications in modern medicine and healthcare. However, their pharmacological mechanisms, clinical efficacy, and potential synergistic effects with other therapeutic agents remain unclear. Further systematic research is needed to clarify molecular targets and optimize their therapeutic applications.

## 1. Introduction

**Figure 1:**
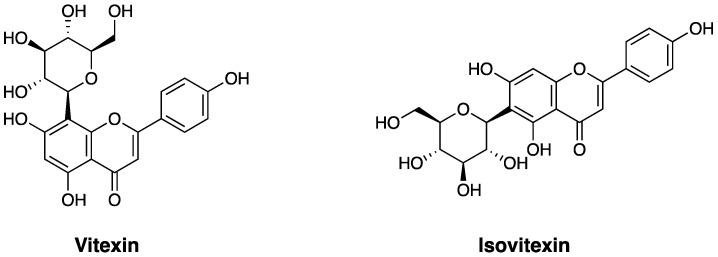
Structure of vitexin and isovitexin.

Vitexin (5, 7, 4-trihydroxyflavone-8-glucoside, Figure 1) is a C-glycosylated flavonoid compound widely present in various kinds of plants and is an active ingredient in many traditional Chinese medicines and foods. Flavonoids often have multiple pharmacological activities; therefore, vitexin has received increasing attention because of its wide range of pharmacological effects, including anticancer, antioxidant, anti-inflammatory, anti-Alzheimer’s disease (AD), blood pressure-lowering, and anti-hypoxia/ischemic injury [1,2,3,4]. These pharmacological effects are related to multiple systems, such as the central nervous, cardiovascular, intestinal system, and endocrine systems [5].

Isovitexin (apigenin-6-C-glucoside, Figure 1), an isomer of vitexin, also exists in plants containing vitexin, such as pigeon peas, passion flowers, bamboo, mimosa, and wheat leaves, and has been screened as a bioactive ingredient [6,7] because of its similar chemical structure. Isovitexin has also been shown to exert pharmacological effects similar to those of vitexin, including antioxidant, anti-inflammatory, and anti-AD effects.

However, a review of the literature reveals several limitations in previous studies. Some works have focused exclusively on vitexin and overlooked isovitexin, while others have addressed both compounds but restricted discussion to individual diseases such as cancer or diabetes, or emphasized isolated domains like extraction processes. Many existing reviews have yet to incorporate recent findings from multiple disciplines, including medicinal chemistry, systems biology, and clinical pharmacokinetics [1,2,3]. These limitations hinder a comprehensive understanding of their therapeutic potential.

To bridge this gap, the present review systematically integrates the recent research on vitexin and isovitexin, spanning from basic pharmacological studies and molecular mechanisms to structural optimization and potential clinical translation. We highlight their roles in treating metabolic diseases, cardiovascular conditions, neurodegenerative disorders, and cancer. This review also emphasizes their complementary pharmacological profiles and structural similarities, aiming to elucidate their synergistic mechanisms and inform future drug development strategies.

This review provides a comprehensive summary of the pharmacological and molecular mechanisms of action of vitexin and isovitexin as well as a brief overview of their pharmacokinetic studies, highlights the potential of vitexin and isovitexin in health prevention and treatment, and proposes future research directions and recommendations, providing a reference for subsequent scientific exploration and clinical applications.

## 2. Methodology

**Figure 2:**
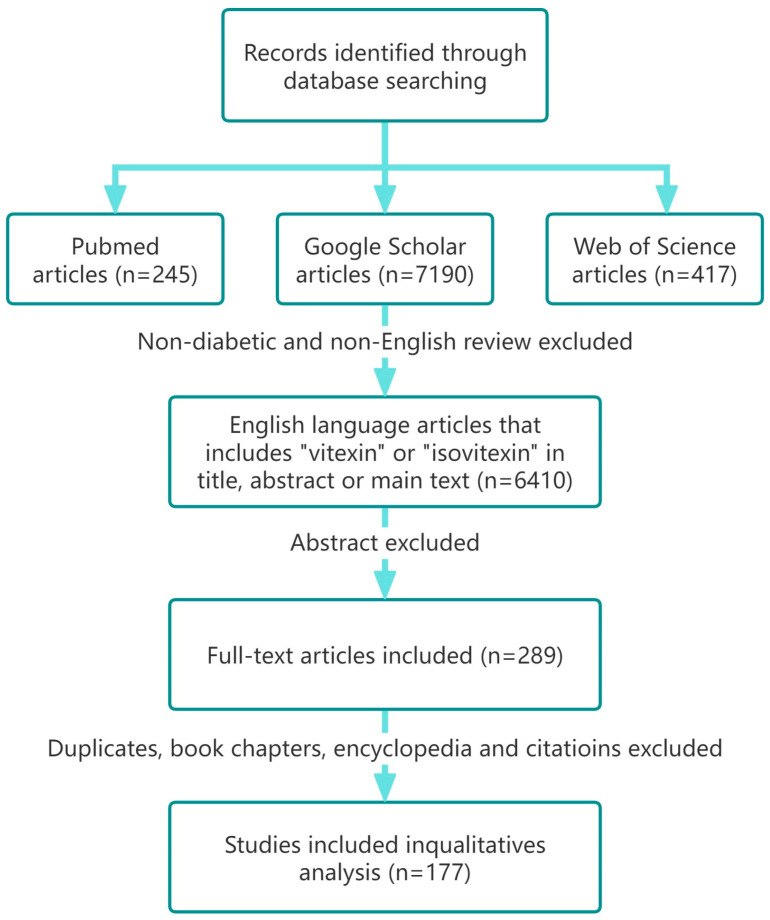
Flowchart of the literature retrieval and screening.

The literature for this study was searched on 10 December 2024, in three electronic research databases: Web of Science (Clarivate Analytics, Philadelphia, PA, USA), Google Scholar (Google LLC, Mountain View, CA, USA), and PubMed (National Library of Medicine, Bethesda, MD, USA). The selection was based on a series of clear inclusion criteria: only research published in English journals must be included, and the keywords “vitexin” and “isovitexin” must be included in the article. The system review excluded review articles, abstracts, editorials/letters, conferences, and conference papers. Articles unrelated to “vitexin” and “isovitexin” in terms of antioxidant, anti-inflammatory, safety, and toxicological applications were also excluded. To further screen the articles, the research results were manually screened to exclude articles that contained duplicate data or were duplicated in the database, as well as some articles on the phytochemical separation of “vitexin” and “isovitexin” derivatives. The remaining articles constituted the main source of information for writing this article. Figure 2 shows the study selection process, through which the researchers ensured the quality and relevance of the included studies to more accurately evaluate the therapeutic potential and health protection of “vitexin” and “isovitexin” in diseases. This systematic approach helps extract the most valuable data from a large amount of work in the literature, providing a scientific basis for further research and clinical applications. Through this process, researchers can provide deeper insights into the use of “vitexin” and “isovitexin” as disease treatment methods and provide guidance for future research.

### Chemical Structure and Sources

Flavonoids are a class of compounds characterized by a 2-phenyl-1-benzopyran-4-one skeleton. Among them, apigenin (4′,5,7-trihydroxyflavone) is a typical natural flavonoid. Vitexin and isovitexin are C-glycosylated derivatives of apigenin, with glucose units attached at different positions—C-8 in vitexin and C-6 in isovitexin. Both compounds share similar chemical structures and properties. Vitexin, also known as 8-D-glucosyl-4′,5,7-trihydroxyflavone or apigenin 8-C-glucoside, has a molecular formula of C_21_H_20_O_10_ and a molecular weight of 432.38 g/mol. Isovitexin, the 6-C-glucoside isomer of vitexin, has the same molecular formula (C_21_H_20_O_10_) and molecular weight (432.38 g/mol), but differs in glycosylation position. Both contain seven hydroxyl groups, and their polyhydroxylated structures contribute significantly to their biological activity. In vitexin, the order of radical scavenging ability among hydroxyl groups is 4′-OH > 7-OH > 5-OH. Additionally, the C-glycoside bond in vitexin is more stable than O-glycosides, which enhances the antioxidant capacity by reducing the negative charge at the C-3 oxygen. C-glycosyl flavonoids generally show stronger antioxidant and antidiabetic properties than their O-glycoside or aglycone counterparts [8].

Vitexin exists in various plants, among which hawthorn leaves are the main source. In recent years, technology for extracting the biological activities of vitexin and isovitexin from plants has been optimized. Compared to the traditional Soxhlet extraction method, there are limitations in solvent selectivity, extraction rate, and toxicity. Microwave-assisted extraction (MAE) is the optimal technique for achieving maximum total polyphenol and flavonoid content and high yield, obtaining high concentrations of antioxidants and anti-inflammatory and antibacterial compounds in a short period of time, with reasonable yield and good selectivity. The preparation and separation of vitexin and isovitexin can be easily and effectively achieved through adsorption and desorption of the ADS-5 resin. This method can also be used for the separation of flavonoid C-glucosides from other Chinese herbal medicines.

## 3. Health Promotion Effects of Vitexin and Isovitexin

**Figure 3:**
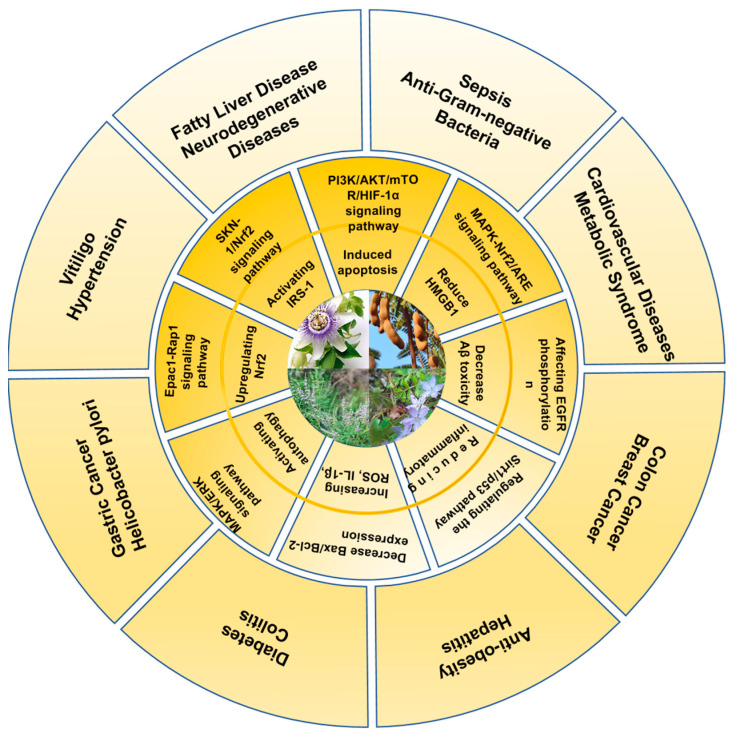
The main mechanisms and treatment types of vitexin and isovitexin.

**Figure 4:**
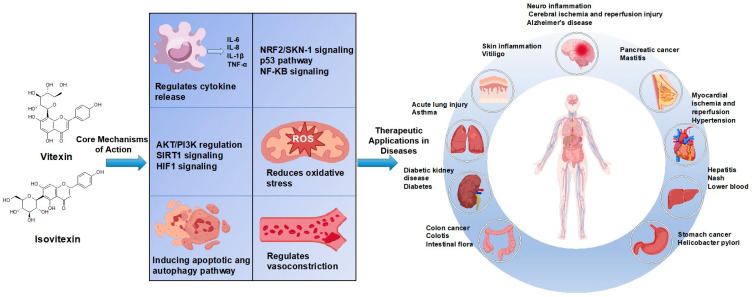
The mechanisms of vitexin and isovitexin and the types of diseases in various systems.

This section highlights the therapeutic effects of vitexin and isovitexin, including cardiovascular protection, blood sugar regulation, anti-obesity, anticancer, antioxidant, anti-inflammatory, and neuroprotective properties. Additionally, they exhibit antimicrobial and antibacterial activities. Table 1 and Figure 3 and Figure 4 illustrate the diseases and treatment types that vitexin and isovitexin can improve, along with their key mechanisms. The yellow inner circle of Figure 3 illustrates the key molecular mechanisms through which vitexin and isovitexin exert their pharmacological effects, while the outer ring shows the corresponding disease conditions or therapeutic areas.

### 3.1. Regulating Cardiovascular Protection

Vitexin has a wide range of pharmacological effects, particularly protective effects on the cardiovascular system. The research has shown that vitexin plays an important role in the cardiovascular system by affecting the myocardium, blood vessels, and platelets through multiple signaling pathways. Vitexin has been shown to have a protective effect against myocardial ischemia–reperfusion injury (MIRI). Experimental data showed that vitexin inhibited the expression of *Epac1* and regulated the Epac1–Rap1 signaling pathway, alleviating MIRI-induced mitochondrial dysfunction and inhibiting the activation of mitochondria-mediated apoptosis pathways, thus exerting a protective effect against MIRI [4]. This discovery provided new targets and a theoretical basis for the potential application of vitexin in the treatment of ischemic myocardial injury. However, this study also has limitations in exploring the specific regulatory mechanism of Epac in ischemia/reperfusion (I/R), and further research is needed to determine the therapeutic potential of vitexin through *Epac1* [9].

Vitexin can also improve the pathological score of the myocardial tissue and reduce the apoptosis index, which is related to its antioxidant activity, inhibition of inflammatory cytokine release, and myocardial cell apoptosis. Experiments have confirmed that doxorubicin (DOX) is an effective chemotherapeutic drug; however, its clinical application is limited by the development of cardiotoxicity. Vitexin exerts a cardioprotective effect against DOX-induced cardiac toxicity by reducing oxidative stress, lowering cardiac inflammatory cytokines, increasing FOXO3a, and inhibiting caspase-3 activation. Therefore, vitexin may serve as an effective therapeutic agent for preventing DOX-induced cardiomyopathy [10,20].

Research has shown that flavonoids, such as vitexin, can regulate vasoconstriction in an agonist-dependent manner, not only through endothelial dependence, but also by inhibiting the MAPK/ERK pathway and partially inhibiting Rho kinase. *Lagenaria siceraria* fruits containing vitexin exhibit anti-hypertensive and anti-cardioprotective effects induced by L-NAME (a NOS inhibitor) [11]. Although further research is needed to determine whether vitexin has anti-hypertensive effects associated with NOS, the existing studies have shown its importance in vascular health.

In summary, vitexin exerts multiple protective effects on the cardiovascular system by affecting the myocardium and blood vessels. These effects include anti-myocardial ischemia/reperfusion injury and anti-vasoconstriction effects, thus playing an important role in the prevention and treatment of cardiovascular diseases. With further research, the cardiovascular protective mechanism of vitexin will be further elucidated, providing a more theoretical basis for clinical applications.

### 3.2. Regulating Blood Sugar and Combating Obesity

Vitexin and isovitexin have shown good therapeutic potential in diabetes as natural compounds with protective effects. The research has indicated that pancreatic injury or pancreatic tissue damage often leads to insufficient insulin secretion or insulin resistance, which in turn affects blood glucose processing and increases blood glucose levels [35,36]. Vitexin has been shown to have a protective effect on pancreatic and islet tissues by upregulating *Nrf2* and antioxidant enzymes, as well as inhibiting MAPK pathways, including c-Jun N-terminal kinase (JNK) and p38, to alleviate the toxicity caused by streptozotocin or lipopolysaccharide (LPS) [37,38].

Oral administration of vitexin and isovitexin can significantly reduce postprandial blood glucose levels in normal blood glucose mice and sucrose-induced diabetic rats. They can also provide potential therapeutic approaches for the prevention of diabetes or other pathological complications through free radical scavenging and metal ion-trapping activities. Simultaneously, α-glucosidase and α-amylase, the key enzymes for carbohydrate digestion, are located on the facial mask on the brush edge of intestinal cells. Vitexin and isovitexin have significant inhibitory effects on α-glucosidase [12,39]. Vitexin and isovitexin were found to exhibit inhibitory effects on AGE formation. For the first time, vitexin and isovitexin were confirmed to inhibit PTP-1B activity, improve insulin signaling, and increase glucose uptake [13]. Research has suggested that acute and long-term hyperglycemia can activate resting endothelial cells and impaired endothelial function of vascular cells. High blood sugar levels induced endothelial damage and apoptosis by reducing the bioavailability of NO and increasing reactive oxygen species (ROS). Vitexin inhibited endothelial activation caused by high glucose levels and reduced vascular inflammation. It also increased superoxide dismutase (SOD) and glutathione peroxidase (GPx) levels and decreased GPX4-mediated ferroptosis. Furthermore, vitexin promoted GLUT4’s translocation from the cytoplasm to the cell membrane, potentially facilitating the uptake of glucose [16,40].

High-dose glucose was added to an in vitro model to create a human umbilical vein endothelial cell (HUVEC) model and examine the protective effect of vitexin against high glucose-induced HUVEC damage. It was demonstrated that vitexin reduced the proliferation and death of HUVECs driven by high glucose, activated Nrf2 in HUVECs, and controlled the Wnt/β-catenin signaling pathway. Vitexin administration simultaneously increased SOD activity and decreased ROS and malondialdehyde (MDA) content in high glucose-induced HUVECs [14].

Obesity-related insulin resistance and glucose metabolism disorders cause cells’ metabolism of fats and carbohydrates to become unbalanced, which eventually results in diabetes. According to the research, instead of depending only on one active ingredient, vitexin and isovitexin are more likely to work in concert to prevent disease. Synergistic gut microbiota regulation may be a component of the pharmacological effects. Insulin resistance in obesity is characterized by decreased insulin-triggered glucose transport and processing in skeletal muscles and adipocytes, as well as inefficient hepatic regulation of glucose synthesis. Vitexin and isovitexin flavonoids not only affected the absorption of peripheral glucose in insulin and non-insulin sensitive tissues but also showed the potential to restore insulin resistance in HepG2 cells by enhancing cellular uptake of glucose. Furthermore, *proteobacteria* are the predominant gut microbiota in obesity and type 2 diabetes, and vitexin and isovitexin have the ability to increase the number of beneficial bacteria while suppressing the levels of harmful bacteria [15].

Through a variety of mechanisms, such as lowering postprandial blood glucose, blocking important digestive enzymes, and controlling intestinal flora, vitexin and isovitexin have demonstrated both preventative and therapeutic effects in diabetes. In addition to being possible medications for atherosclerosis and cardiovascular problems in diabetes, vitexin and isovitexin may offer novel approaches for the prevention and management of vascular diseases. Vitexin and isovitexin can be used as functional ingredients in the industrial food sector. To clarify the metabolic pathways of bacteria and understand the specific microbial glucose metabolism process, more research is required. Additional investigation of the distinct routes of action of these chemicals will offer a scientific foundation for the development of novel treatment approaches.

Adipose tissue abnormalities and excessive accumulation are intimately linked to inflammation, as evidenced by elevated TNF-α and IL6 release. Vitexin dramatically decreased serum levels of IL6 and TNF-α in *C57BL/6J* mice fed a high-fat diet. Fat formation is inhibited when AMPK α, the primary regulator of energy metabolism, is phosphorylated [17,18,27]. Previous studies have shown that vitexin ameliorated weight gain and obesity induced by a high-fat diet (HFD) in *C57BL/6J* male mice, inhibited adipogenesis in adipocytes, dramatically increased the expression of phosphorylated AMPK α, and decreased the expression of C/EBP α and fatty acid synthase (FAS) in white adipose tissue and 3T3-L1 adipocytes [41].

Vitexin-induced decrease in adipogenesis may involve additional signaling pathways besides AMPK alpha. Vitexin has been shown to reduce the inflammation and apoptosis induced by ER stress by blocking adipogenesis through ER stress-induced pathways. Conversely, vitexin prevents HFD-induced vasculitis by blocking TMAO-mediated RNA N6-methyladenosine (m6A) alteration, which paves the way for the creation of functional foods [42].

In conclusion, vitexin decreased the expression of adipogenic genes in white adipose tissue and relieved obesity induced by a high-fat diet [41]. Further research is necessary to determine the possible impacts of vitexin on the upstream regulators of the AMPK α and ER pathways. Vitexin could therefore be another dietary supplement that can be utilized in conjunction with other methods for treating and preventing obesity and its associated problems.

### 3.3. Lowering Blood Cholesterol and Treating Fatty Liver

Although liver damage from chronic alcohol use is inevitable, few effective liver-protective medications are currently available to treat ethanol-induced liver damage. The development of alcoholic liver disease (ALD) is significantly influenced by apoptosis associated with liver injury. Hepatitis can result from the discharge of apoptotic cells into the extracellular space, which can cause inflammation if they are not quickly removed. Furthermore, the phagocytic action of apoptotic cells has a pro-fibrotic effect, and they also release pro-fibrotic mediators, which cause fibrosis of liver cells. Thus, ALD can be slowed or reversed by blocking apoptosis [43]. Studies have demonstrated that vitexin possesses antiapoptotic properties, preserves the structural integrity of liver cells, controls lipid metabolism, and dramatically reduces ethanol-induced LO2 cell damage. The protective effect of vitexin against ethanol-induced liver damage was closely linked to the Sirt1/p53-mediated apoptosis pathway, as evidenced by the fact that vitexin inhibited serum TG and TC levels and significantly improved ethanol-induced ALD [44], and its protective effect against ethanol-induced liver injury was closely linked to the Sirt1/p53-mediated apoptosis pathway, as Sirt1 could remove the acetyl group of p53, reduce the transfer of p53 from cytoplasm to mitochondria, inhibit the release of mitochondrial pro-apoptotic proteins, and ultimately restrict apoptosis [45].

Currently, there is no approved treatment for non-alcoholic fatty liver disease (NAFLD), a prevalent liver illness characterized by the accumulation of liver fat. Since diabetes and obesity are the primary causes of NAFLD, substances with anti-obesity properties and the ability to lower insulin resistance are regarded as promising candidates for the treatment of NAFLD [46]. Studies have demonstrated that vitexin can decrease the body and liver weight of *C57BL/6J* mice fed an HFD, as well as the serum and liver levels of cholesterol and triglycerides. In the HFD group, vitexin markedly decreased the serum levels of aspartate aminotransferase (AST) and alanine aminotransferase (ALT). Activating receptor gamma (PPAR), FAS, steroid regulatory element binding protein-1c (SREBP-1c), CCAAT/enhancer binding protein alpha (C/EBPα), peroxisome proliferators, and acetyl CoA carboxylase (ACC) were also downregulated by vitexin, which inhibited lipogenesis. By activating insulin receptor base-1 (IRS-1) and its downstream target AKT, vitexin enhances insulin signaling [17,47]. NAFLD may worsen because of chronic stress (CS). Research has demonstrated that vitexin helps treat NAFLD caused by a high-fat diet and chronic stress. This mechanism is directly linked to lowering the accumulation of fatty acids and suppressing the TLR4/NF-κ B signaling pathway. Vitexin addresses lipid metabolic issues, reduces liver inflammation, and decreases the accumulation of fat in the liver. In addition, vitexin suppresses the expression of proteins and genes linked to the production of fat in the liver [2].

The initial hypothesis was that isovitexin prevents liver fibrosis. Isovitexin could alleviate liver fibrosis in two hepatic stellate cell models induced by platelet-derived growth factor BB (PDGF-BB) and in mouse liver fibrosis models induced by carbon tetrachloride (CCl_4_) by reducing collagen deposition and hepatic stellate cell activation. According to transcriptomic and metabolomic analyses, the primary regulatory processes of isovitexin in liver fibrosis may be the phosphatidylinositol 3-kinase (PI3K)/protein kinase B (Akt) signaling pathway and the glutathione (GSH) metabolic pathway, also confirming that *miR-21* is a crucial node in the isovitexin regulation of the PI3K/Akt signaling pathway and GSH metabolic pathway [19].

The biological roles of vitexin and isovitexin, which are possible medications for the treatment of fatty liver disease, may include immune control, lipid metabolism regulation, and anti-inflammatory activities. These results place vitexin and isovitexin as a solid scientific foundation for novel medications for fatty liver and offer evidence in favor of further clinical studies and development.

### 3.4. Anticancer Effect

As the second greatest cause of mortality and one of the main global public health concerns, cancer not only endangers lives, but also has a significant financial impact. Thus, creating successful plans for long-term cancer prevention and control is essential [48]. Plant-derived secondary metabolites have shown beneficial anticancer properties. Studies conducted both in vitro and in vivo have demonstrated that the chemopreventive chemicals vitexin and isovitexin exhibit pharmacological efficacy against a variety of malignancies by boosting autophagy or apoptotic processes and preventing migration and proliferation [1]. According to the preliminary research, vitexin and isovitexin demonstrated antitumor activity through targeted cell apoptosis in HepG2 hepatocytes, HeLa cervical cells, MCF-7 human breast cancer cells, U937 human leukemia cells, and HCT116 colorectal cells [49,50,51,52].

Changes in the pathological milieu from an inflammatory state to a pro-tumor microenvironment are key risk factors for the development of colitis-associated cancer (CAC), which is caused by chronic colitis. The deadly effects of inflammatory cytokines generated by M1 macrophages were maintained by vitexin’s ability to bind to the vitamin D receptor (VDR) protein, which inhibits macrophage development towards the M2 phenotype. In vitro co-culture studies have shown that vitexin enhances the anticancer activity of M1 macrophages by sustaining their pro-inflammatory phenotype and inhibiting the M2 polarization typically associated with tumor promotion. The targeting effect of vitexin on VDR was further validated using myeloid-specific VDR gene knockout mice. The shift from colitis to colorectal cancer is a major pathogenic role that VDR inhibits [53,54]. The important role of VDR as a nuclear receptor transcription factor in colitis and colorectal cancer has been confirmed by clinical and basic research. Isovitexin has been found to activate the p53 signaling pathway in a dose-dependent manner, thereby exerting inhibitory effects on colorectal cancer cells. The possible use of isovitexin in the treatment of colorectal cancer now has a scientific foundation for this discovery [23].

Gastric cancer (GC) is a serious threat to public health and its high recurrence rate after surgery is the main reason for the short survival of patients. The primary cause of stomach cancer recurrence is metastasis, and epithelial–mesenchymal transition (EMT) is a crucial step in the metastatic process. Two distinct gastric cancer cell lines (SGC-7901 and AGS) and a normal, non-transformed gastric cell line (GES-1) were used to assess the antitumor potential of vitexin, a medication that is thought to be a contender for the treatment of gastric cancer. These findings showed that vitexin had a dose-dependent effect on gastric cancer cells’ production of HIF-1 α. Additionally, by deactivating the PI3K/AKT/mTOR/HIF-1 α pathway, vitexin therapy slowed the growth of gastric cancer cells. In particular, it was discovered for the first time that vitexin inhibits the proliferation, migration, invasion, and EMT of gastric cancer cells by downregulating the expression of HMGB1 in these cells. More significantly, vitexin inhibited the activation of the PI3K/AKT/mTOR pathway by promoting the nuclear translocation of HMGB1. According to the study results, vitexin might be a novel and potent medication for the treatment of stomach cancer. Nevertheless, it is still unknown how vitexin inhibits HMGB1 in gastric cancer cells [21].

According to the research, vitexin can cause cell death by triggering apolipoprotein L1 (ApoL1)-mediated autophagy and JNK and suppresses the activity of heat shock factor 1 (HSF-1) to prevent the growth of colorectal cancer cells [53]. Many cancers overexpress cyclin-dependent kinases (CDKs), which are important targets of new anticancer medications. According to experimental findings, vitexin’s IC_50_ value for HCT116 colorectal cancer cells was 203.27 ± 9.85 μ mol/L. According to Zhao et al. (2024) [22], vitexin may have an inhibitory effect on colorectal cancer cell proliferation, G2/M conversion, cell cycle progression, and CDK1/cyclin B protein production in HCT116 cells.

Triple-negative breast cancer (TNBC) is a type of breast cancer with a dismal prognosis and is insensitive to targeted and endocrine therapies. Following chemotherapy, it has a poor prognosis and significant recurrence rate. Tumor-associated macrophages (TAMs) are essential for the development of TNBC. According to previous studies, vitexin can stop TNBC cells from proliferation and invading, and can also prevent the migration of cancer cells. Furthermore, vitexin can influence the downstream signaling and phosphorylation of the epidermal growth factor receptor (EGFR), block M2 polarization, enhance M1 macrophage polarization, and exert anticancer effects by targeting the EGFR/PI3K/AKT/mTOR pathway [20,55]. When combined with doxorubicin (Dox), vitexin can reduce tumor growth and show synergistic effects in in vivo tests, increasing antitumor efficacy. However, more research is needed to determine how vitexin controls this pathway and how it affects macrophage polarization. Furthermore, there is potential for the therapeutic application of vitexin’s effect on other immunological targets in breast cancer, which has yet to be investigated.

Chemotherapy remains the principal treatment for patients with non-small cell lung cancer (NSCLC), although anticancer medications frequently have negative side effects. Therefore, finding novel, low-toxicity anti-NSCLC medications is crucial. The possible antitumor action of vitexin in NSCLC has been verified in previous studies. Treatment with vitexin and isovitexin decreased the A549 cells in vitro survival rate and lessened the increase in lactate dehydrogenase (LDH) release caused by cell membrane disruption. Further evidence of the anti-NSCLC potential of vitexin came from the administration of both vitexin and isovitexin, which both suppressed the growth of NSCLC tumors in vivo. Based on the findings of this study, vitexin and isovitexin lowered the Bcl-2/Bax ratio and caused cytochrome c to be released from the mitochondria into the cytoplasm, which cleaved caspase-3 in A549 cells. It was hypothesized that vitexin uses a mitochondrial-dependent pathway to partially trigger apoptosis in A549 cells. Through in vitro and in vivo experiments, the survival rate of A549 cells damaged by vitexin treatment was revealed for the first time, and apoptosis was partially induced through the mitochondrial pathway and the PI3K/Akt/mTOR signaling pathway. Vitexin and isovitexin are expected to be innovative and effective drugs for the treatment of NSCLC [24,25].

Through controlling the expression of *miR-34a*, isovitexin was shown to suppress the stem cell properties of SK-Hep-1 cells. Isovitexin decreased the number of CD44+ cells and prevented the development of spheroids and colonies. Furthermore, isovitexin showed promise in treating liver cancer by increasing the amount of Bax protein in SK-SC cells and lowering the levels of Bcl-2 and Mcl-1 proteins, which are linked to apoptosis. This provides isovitexin’s new approach to liver cancer treatment as a scientific foundation, particularly its regulatory effect on the traits of liver cancer stem cells, which could serve as crucial targets for the development of novel therapeutic approaches [26].

Vitexin and isovitexin have antitumor properties through a number of mechanisms, such as triggering cell death, preventing tumor growth, inhibiting tumor cell invasion and migration, regulating immune responses and tumor cell autophagy, and directly suppressing tumor development in specific cancers. These mechanisms provide a theoretical basis for exploring vitexin and isovitexin as promising candidates for anticancer drug development.

### 3.5. Antioxidant Effect

Oxidative stress is a harmful process at the cellular or subcellular level and may become an important mediator of damage to cellular structures and various disease states. Reactive oxygen species-induced lipid peroxidation produces lipid peroxides in the cell membrane, causing extensive damage to the membrane structure and membrane-mediated chromosomal damage [56]. In recent years, vitexin and isovitexin have shown good antioxidant properties in both in vitro and in vivo studies.

Excessive nitrosation and oxidative stress can trigger inflammatory responses, leading to an increase in inflammatory mediators, which subsequently activate apoptotic pathways, are crucial for neuronal cell death, and play an important role in the development of hyperactivity disorder (OD) [38]. In vitro experiments confirmed for the first time that the flavonoid compound vitexin protects against neuro-inflammation, nitrosation, oxidative damage, mitochondrial dysfunction, apoptotic activation through the Nrf2 pathway, and haloperidol-induced hyperactivity disorder (HPD-OD). According to the study, the antioxidant qualities of vitexin were ascribed to its capacity to directly scavenge oxygen free radicals and shield antioxidant enzymes. Vitexin is a potent free-radical scavenger because it can supply electrons via the nearby dihydroxy structure of the A ring. Vitexin also increased *Nrf2* expression and boosted GSH and antioxidant enzymes such as SOD, CAT, GPx, and GST. Additionally, vitexin can exert anti-inflammatory effects by inhibiting NF kB transcription factors, downregulating pro-inflammatory mediators such as TNF-α and IL-1 β, and upregulating anti-inflammatory mediators such as IL-4 and IL-10 [29,57].

Vitiligo is a common skin depigmentation disease, and its pathogenesis is related to cellular oxidative stress caused by reactive oxygen species (ROS). Vitexin has been shown to inhibit the apoptosis of melanocytes caused by H_2_O_2_, promote cell proliferation, reduce the expression of inflammatory factors and ROS, and upregulate the expression of antioxidant genes HO-1 and SOD by activating the MAPK-Nrf2/ARE signaling pathway, thereby exerting its antioxidant effects. When Nrf2 was knocked down, the protective effect of vitexin was reversed, indicating that Nrf2 plays a key role in the antioxidant effect of vitexin. This study not only emphasizes the importance of the Nrf2/ARE axis in the antioxidant defense of melanocytes but also provides new strategies for the treatment of vitiligo [27].

Numerous pathophysiological processes such as tumor development, aging, inflammation, diabetes, and neurological illnesses are significantly affected by DNA damage. DNA damage results from highly active endogenous aldehydes produced by cellular processes such as oxidative stress, lipid peroxidation, and glycosylation. These aldehydes directly react with DNA to form DNA aldehyde-derived adducts. Mice that drink diluted alcohol suffer harm to their hematopoietic stem cell DNA as a result of the metabolism of ethanol into acetaldehyde. Cellular homeostasis problems caused by unrepaired DNA damage can accelerate the development and accumulation of the disease phenotypes. Different doses of vitexin have been shown to have antioxidant effects that can repair ethanol-induced DNA damage, suggesting its possible use in the treatment of liver injury [58].

In *Caenorhabditis* elegans, studies have shown that vitexin and isovitexin, as putative SKN-1/Nrf2 activators, increase lifespan and support a healthy lifespan. To eliminate ROS generated under stress, vitexin and isovitexin increased the expression of antioxidant genes and proteins, decreased the accumulation of ROS, and increased the accumulation of SKN-1 in the nucleus. Vitexin and isovitexin have shown promise as prospective compounds for novel approaches in the chemoprevention of aging and oxidative-related disorders, and they offer fresh perspectives on activating the SKN-1/Nrf2 pathway [59].

By lowering intracellular ROS levels, suppressing the expression of heat shock proteins (Hsp70 and Hsp90), and shielding cells from the synthesis of apoptotic proteins, vitexin can effectively prevent heat stress-induced cell death. In addition to offering possible application opportunities for the management of people working in hot environments and patients suffering from heat-related illnesses, this discovery provides a fresh perspective for the prevention and treatment of heat stress [60]. Research has found that vitexin inhibits ROS production by promoting PPARγ activity, enhancing antioxidant enzyme activity [30].

Chronic kidney disease (CKD) is a systemic inflammatory syndrome characterized by tubulointerstitial inflammation. LPS, an outer membrane component of Gram-negative bacteria, increases the production of ROS and triggers cellular inflammation. Isovitexin has been demonstrated in vitro to improve cell viability in SV40-MES-13 cells and inhibit ROS generation generated by LPS. Isovitexin also decreased inflammatory and apoptotic markers and enhanced the mitochondrial membrane potential. Isovitexin decreased kidney damage and inflammation in a *C57BL/6* mouse kidney injury model by enhancing protective autophagy, reducing ROS generation, and exhibiting anti-inflammatory and anti-pyroptotic properties. Isovitexin also regulates the NF-κB, P53, PI3K/Akt, and MAPK signaling pathways, which can cause autophagy in HepG2 human liver cancer cells. These findings suggest that isovitexin may exert a protective effect against kidney injury through multiple mechanisms [22,32].

Both vitexin and isovitexin demonstrated antioxidant activity both in vitro and in vivo. They achieve this by directly inhibiting oxidative stress markers, reducing inflammation, encouraging autophagy, and shielding endothelial cells. These processes complement each other, making them promising candidates for antioxidant medications. These findings offer a solid scientific foundation for investigating the antioxidant mechanisms and the possible uses of isovitexin and vitexin.

### 3.6. Anti-Inflammatory Effect

Vitexin has demonstrated possible protective effects against autoimmune hepatitis (AIH), a chronic progressive liver disease [3]. According to previous studies, taking vitexin dramatically lowered serum ALT and AST levels, reduced oxidative stress-induced liver damage, and decreased the infiltration of inflammatory cells and CD4 + T cells into the liver. Furthermore, vitexin ameliorated liver damage in EAH animals by upregulating the *Nrf2* gene and activating the AMPK/AKT/GSK-3 β pathway, offering scientific support for vitexin as a novel and potent medication for AIH treatment [29].

Several inflammatory cytokines, including IL-6, TNF-α, and IL-8, are associated with skin inflammation, aging, and photoaging. Following UV exposure, these cytokines increase and affect the epidermal barrier function. The excessive generation of ROS and reactive nitrogen species (RNS) is also linked to inflammation, which accelerates the development of wrinkles and skin dryness. According to previous research, vitexin has anti-inflammatory properties and helps treat skin irritation; however, it has no discernible effect on TNF-α levels and may instead control keratinocyte differentiation rather than death. Through free radical scavenging and anti-inflammatory mechanisms in primary human skin cells, vitexin was shown to eradicate UV-induced ROS/RNS for the first time [61].

Since Nrf2 is thought to be a desirable biological target for vascular inflammation, it is possible to create small-molecule medicines that specifically target it to treat metabolic disorders. Non-covalent inhibitors may be less hazardous than covalent Nrf2 activators for Nrf2 activation [62]. Vitexin is absorbed by HUVECs in a dose-dependent manner. Vitexin inhibits vascular inflammation by directly binding to the Kelch domain of Keap1, breaking the Keap1-Nrf2 connection, and releasing Nrf2 for transport to the nucleus, highlights a new function for Nrf2 in vascular inflammation and offers intriguing molecular pathways for innovative approaches to treating metabolic and cardiovascular disorders [28,63].

Lipopolysaccharide (LPS) is a microbial toxin that is one of the main causes of sepsis. During inflammation, LPS is known to impair the function of P-glycoprotein (P-gp), and molecular docking studies have shown that vitexin is an effective substrate for P-gp, while verapamil is an effective inhibitor of P-gp. Mice were intraperitoneally injected with LPS (10 mg/kg), and macrophages showed increased levels of H_2_O_2_, superoxide, and NO, whereas antioxidant activity decreased. Mice treated with vitexin (5 mg/kg body weight) and verapamil (5 mg/kg body weight) showed higher antioxidant enzyme activities (SOD, CAT, and GRx), thereby reducing oxidative stress. This combination therapy simultaneously downregulated the expression of TLR4, NF-κB, and P-gp in mouse peritoneal macrophages, leading to a polarization transition from M1 to M2 macrophages and reduced inflammatory responses. Therefore, combined therapy with vitexin and verapamil may be a potential therapeutic strategy to shield the body from inflammatory damage and sepsis caused by LPS [64].

A study using an LPS/D-GalN-induced acute liver injury (ALI) animal model found that isovitexin has potential therapeutic effects in ALI. Treatment with isovitexin dramatically decreased the total m6A and m6A modification levels of phosphatase and tensin homolog (PTEN) and binding immunoglobulin protein (BiP) in the liver, while upregulating the expression of PI3K, Akt, and mTOR. PTEN is a negative regulator of the PI3K/Akt pathway and plays a crucial role in controlling immune cell activation and pro-inflammatory signaling. BiP, an endoplasmic reticulum (ER) chaperone, is a key indicator of ER stress, which is often linked to inflammation and cell apoptosis. These findings suggest that isovitexin may alleviate liver inflammation by modulating PTEN and BiP expression through m6A modification, thereby influencing key inflammatory signaling cascades. To further validate these mechanistic insights, another study demonstrated that isovitexin treatment reduced the inflammatory response induced by lipopolysaccharide (LPS) in both in vitro macrophage models and an in vivo LPS-induced mouse ALI model, highlighting its potential as a novel therapeutic agent for acute liver injury [65].

Ulcerative colitis (UC) is an idiopathic inflammatory disease of the colonic mucosa. Studies have shown that dextran sulfate sodium (DSS) administration can cause chemical damage to the intestinal epithelium of mice, leading to colon atrophy and inflammation. Vitexin treatment significantly inhibited colonic atrophy induced by DSS in colitis mice and reduced colonic mucosal inflammation, ulceration, and necrosis [66,67]. Vitexin reduced inflammation and DSS-induced colon damage by concurrently blocking the phosphorylation of the p65, I κ B, and STAT1 proteins in colon tissue. In addition to its anti-inflammatory properties, the ability of vitexin to alter the colonic mucosal barrier is another crucial molecular mechanism in the fight. The gut microbiota is vital for controlling the function of the intestinal mucosal barrier and the inflammatory response, and there is mounting evidence that it plays a significant role in the onset and progression of colitis. Increased intestinal permeability and inflammatory activation brought on by gut microbiota dysfunction can hasten the onset of colitis [68,69]. Therefore, regulation of the gut microbiota is considered an important strategy for the treatment of UC. Research has found that vitexin regulated the gut microbiota of DSS-induced colitis mice and significantly increased the abundance of protective symbiotic bacteria, revealing that vitexin can alleviate colitis by reversing dysbiosis of the gut microbiota in DSS-induced colitis mice. In summary, vitexin exerts significant therapeutic effects in mouse models of DSS-induced colitis by suppressing intestinal mucosal inflammation, preserving intestinal barrier integrity, and reversing gut microbiota dysbiosis associated with colitis. In addition, vitexin has been shown to significantly improve chronic colitis symptoms in CAC mouse models induced by azoxymethane and dextran sulfate sodium (AOM/DSS), alleviate colon damage, and regulate inflammatory cytokine levels in the colon [53]. Therefore, vitexin may be a potential drug for treating or preventing colitis and provides a scientific basis for potential therapeutic drugs to prevent the progression of colitis to colorectal cancer [15,57].

Mastitis is caused by several pathogenic microorganisms. *Staphylococcus aureus*-induced mastitis produces a large number of DEGs, including immune signaling pathways, apoptosis, and endoplasmic reticulum stress. Vitexin may also reduce inflammatory factors and apoptosis by alleviating endoplasmic reticulum stress and inactivating the MAPK and NF-κB signaling pathways. Vitexin may have great potential as a preventive and therapeutic agent for mastitis [30].

Allergic contact dermatitis (ACD) is a skin disease caused by environmental and occupational allergens. During the sensitization stage of ACD, allergens activate innate immunity by releasing pro-inflammatory cytokines and chemokines through keratinocytes, recruiting T cells, and producing cytokines, such as TNF-α, IFN-γ, and IL-17A. Appropriate immune regulation is of great significance in preventing ACD. A study optimized an ACD mouse model induced by *Ginkgo biloba* acid (GA). This study showed that isovitexin reduced MAPK phosphorylation, decreased NF-κB activation, and promoted the M2 polarization of macrophages. It was also found that isovitexin blocked SHP2 with immunomodulatory effects, dose-dependently upregulated apoptosis, and inhibited cytokines, such as TNF-α, IFN-γ, IL-2, and IL-17A [70].

When the serum uric acid level in patients with gouty arthritis (GA) increases, supersaturated urate salts precipitate to form crystals and deposit in the synovial tissue. These urate crystals are recognized by TLR4 and activate the TLR4 signaling pathway, leading to the transcription, synthesis, and release of downstream pro-inflammatory factors, causing acute inflammatory reactions in joints. Research has found that isovitexin attenuated the recognition and internalization of TLR4 in inflammatory responses, blocked intracellular transmission of MyD88 connections, and inhibited the activation of NF-κB. In addition, treatment with isovitexin + TLR4 inhibitors could inhibit the release of related inflammatory factors [31].

Vitexin and isovitexin exert their anti-inflammatory activity through various mechanisms, including regulating immune responses and inflammatory cytokines, activating the Nrf2 pathway, affecting LPS-induced inflammatory responses, and exhibiting good anti-inflammatory effects on colitis, skin inflammation, and mastitis. The discovery of these anti-inflammatory mechanisms provides a scientific basis for the application of vitexin and isovitexin in the treatment of metabolic diseases.

### 3.7. Neuroprotective Effect

Vitexin and isovitexin have various pharmacological properties, including neuroprotective effects. By reducing the levels of pro-inflammatory and cytotoxic factors in LPS-induced microglia and mediating the NF-κB signaling pathway, neuroprotective effects have been demonstrated [33,71]. Research has shown that vitexin can regulate cell activity through the AKT/mTOR, p53, or Bcl-2/Bax pathways. In the treatment of cerebral ischemia–reperfusion injury (CIRI), vitexin can improve the behavior of CIRI mice and inhibit oxidative damage in the mouse brain, thereby showing its potential as a drug candidate for stroke treatment [72].

The short-term and long-term neuroprotective effects of vitexin were assessed by using a rat neonatal model. Vitexin, as an HIF1-α inhibitor, has demonstrated good protective effects by decreasing neuronal damage and reducing infarct volume, particularly when administered early after hypoxic–ischemic (HI) injury. Moreover, vitexin reduced cerebral edema, which is linked to the downregulation of VEGF and HIF-1-α, and helped preserve the integrity of the blood–brain barrier (BBB). It has been demonstrated that vitexin protects against a cerebral ischemia/reperfusion (I/R)-induced increase in the permeability of brain endothelial cells [34]. Vitexin decreased inflammation and LDH and caspase 3 levels in the human brain microvascular endothelial cells (HBMEc) I/R damage model. Additionally, vitexin suppressed matrix metalloproteinase (MMP) activity and increased the expression of tight junction proteins [46].

Research has shown that vitexin has a protective effect on cerebral ischemia–reperfusion injury by upregulating p-ERK1/2, downregulating p-JNK and p-P38, increasing Bcl-2, and decreasing Bax expression in the cortex and hippocampus. The data suggested that vitexin may exert its effect by regulating the mitogen-activated protein kinase (MAPK) signaling pathway [73].

In summary, vitexin, as a neuroprotective agent, may regulate multiple signaling pathways, including inhibition of HIF-1-α, modulation of the MAPK signaling pathway, and impact on the expression of apoptosis-related proteins. In addition, the neuroprotective effect of vitexin may be achieved through other pathways and targets, such as reducing free radical levels, combating neuronal apoptosis, regulating inflammatory factors and their pathways, and modulating neurotransmitters and related receptors. Vitexin has a significant protective effect on the increased permeability of brain endothelial cells caused by cerebral ischemia/reperfusion through various mechanisms, including the reduction in cell damage markers, alleviation of inflammatory responses, maintenance of the integrity of the blood–brain barrier, and regulation of nitric oxide synthase activity. These findings provide scientific evidence that vitexin is a potential neuroprotective agent. This may also provide new strategies for the treatment of cerebral ischemia–reperfusion injury in the future.

Another important neuroprotective role of vitexin has been demonstrated through its potential role in Alzheimer’s disease (AD). AD is a chronic neurodegenerative disease characterized by the deposition of the beta amyloid protein (Aβ) to form senile plaques. Research using a *Caenorhabditis* elegans model of AD has shown that vitexin treatment can significantly prolong the lifespan of nematodes and delay Aβ-induced paralysis, which may be related to its antioxidant activity. In nematodes, *acetylcholinesterase gene 1* (*ace-1*) and *acetylcholinesterase gene 2* (*ace-2*) together account for approximately 95% of total acetylcholinesterase activity and are critically involved in the termination of cholinergic neurotransmission. Vitexin treatment significantly inhibited acetylcholinesterase activity and markedly downregulated the expression of *ace-1* and *ace-2*. In addition, it conferred neuroprotection against Aβ-induced toxicity in nematodes and upregulated the expression of the acr-8 gene. These findings suggest that vitexin, as a multifunctional compound, holds promise as a potential therapeutic agent for the treatment of Alzheimer’s disease [74,75].

### 3.8. Antimicrobial and Antibacterial Effect

Vitexin and isovitexin have also shown promising potential in modulating intestinal microbiota and in turn play a significant role in regulating various diseases such as overweight [15], acute colitis [67], neuro-inflammation [57], and lipid metabolism disorders [76]. The gut microbiota is a key mediator of health, influencing a wide range of physiological processes such as metabolism, immune response, and inflammation [68,69]. Recent studies have suggested that vitexin and isovitexin exert therapeutic effects by altering the composition of gut microbiota, thereby mitigating disease symptoms.

Research has shown that vitexin has inhibitory effects on Gram-negative bacteria, including *Proteus mirabilis*, *Escherichia coli*, and *Enterobacter cloacae*, and even exhibits antibacterial effects against *Pseudomonas aeruginosa*, which has the potential to resist biofilms [77,78]). Vitexin can also resist *Helicobacter pylori* infection, which may be related to its anti-myeloperoxidase (MPO) enzyme activity and inhibition of H- and K-ATPase activity [79,80].

In terms of antiviral activity, vitexin is resistant to rotavirus and parainfluenza type 3 virus but has relatively weak resistance to human immunodeficiency virus (HIV). Although the molecular mechanism of its antiviral activity is still unclear, vitexin has shown resistance to plant viruses such as *Tobacco mosaic* virus (TMV) [81].

Vitexin’s anti-inflammatory and antioxidant properties, as well as its inhibitory effect on a variety of pathogens, have potential applications in the treatment of mastitis and other infectious diseases [30], indicating that vitexin may become a new anti-infective drug. In a human gut simulation model, both vitexin and isovitexin were shown to affect the abundance of several gut bacterial genera, including *Adlercreutzia*, *Akkermansia*, and *Streptococcus*. These changes were associated with a reduction in the risk of T2DM in overweight individuals. In an acute colitis mice model, vitexin significantly regulated the abundance of beneficial gut bacteria such as *Veillonella*, *Parabacteroides*, and *Flavonifractor*, which are associated with reduced gut inflammation. This modulation of gut microbiota was linked to the alleviation of colitis symptoms, supporting the potential of vitexin as a therapeutic agent for inflammatory bowel diseases. Vitexin has been shown to exert neuroprotective effects by modulating the gut microbiota in a mice model of neuro-inflammation. This research suggests that vitexin’s ability to regulate gut bacteria contributes to its neuroprotective effects, potentially offering a novel approach for treating neurodegenerative diseases. In a study of lipid metabolism disorders, vitexin was found to influence the levels of *Rombousia* and *Faecalibaculum* bacteria, both of which play a role in regulating lipid metabolism. This highlights the importance of gut microbiota modulation in addressing conditions related to dyslipidemia and obesity.

These findings demonstrate that vitexin and isovitexin can influence a broad range of diseases through their ability to modulate the intestinal microbiota. This emerging area of research suggests that these flavonoid compounds not only exert direct pharmacological effects but also influence disease processes indirectly via the gut microbiota, offering potential therapeutic benefits for a variety of conditions.

## 4. Mechanisms

**Figure 5:**
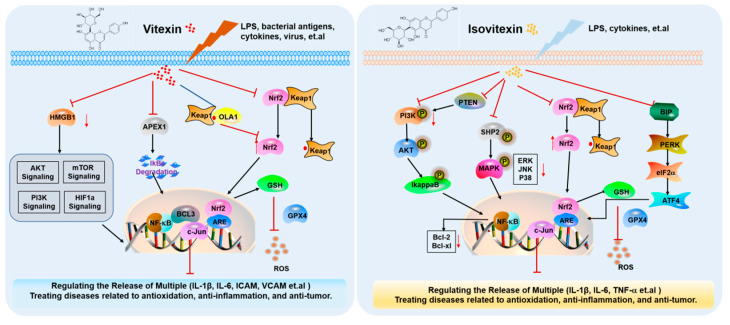
Target mechanism diagram of vitexin and isovitexin.

To address the potential redundancy suggested in Section 3 and to enhance clarity, we have summarized the core mechanisms of vitexin and isovitexin in Table 2 and Figure 5. This table consolidates the major pharmacological effects alongside their corresponding molecular pathways and target molecules, providing a clear and systematic overview. Rather than repeating detailed mechanistic descriptions, this section highlights the key molecular targets and regulatory axes that have been consistently observed across different disease contexts, thereby facilitating a more integrated understanding of the bioactivities of vitexin and isovitexin.

Building upon the summarized evidence, we further elaborate on the biological activities and molecular functions of vitexin and isovitexin in this section. In Table 2, these compounds have demonstrated antioxidant, anti-inflammatory, anticancer, neuroprotective, and cardioprotective properties. Additionally, they have demonstrated lipid-lowering effects, regulation of glucose metabolism, and hepatoprotective activity. Both in vitro and in vivo studies have validated their anticancer potential, primarily through the induction of apoptosis and autophagy [25,26]. Consequently, the molecular targets and therapeutic relevance of vitexin and isovitexin in disease treatment have attracted significant research interest.

Nrf2 is widely regarded as an attractive biological target, and small-molecule compounds that activate Nrf2 can be developed for the treatment of metabolic diseases. Research has confirmed that vitexin significantly enhances the expression of the Nrf2 protein. Using tissue adenosine triphosphate (TPP), molecular docking, siRNA-mediated RNA interference, and in vitro cell-based studies, Obg-like ATPase 1 (OLA1) was reported for the first time as a novel protein target of vitexin that activates Nrf2 and inhibits colitis through non-covalent interactions with Keap1. Vitexin significantly reduced the elevated levels of TNF-α and IL-1 β in the serum of DSS-induced colitis. In addition, after treatment with vitexin, the levels of pro-inflammatory cytokines (IL-1 β, IL-6, ICAM, and VCAM) in the colon of DSS-induced colitis mice were significantly reduced at both the gene and protein levels [40,63,82]. OLA1 is a novel target protein that contributes to the anti-inflammatory effects by increasing the Nrf2 protein expression. OLA1 provides a molecular mechanism for a new therapeutic strategy for colitis and related systemic inflammations [82].

Vitexin has therapeutic effects on chronic kidney disease (CKD), a progressive and destructive disease that may ultimately lead to irreversible renal failure, end-stage renal disease (ESRD), and premature death. Previous studies have confirmed that vitexin protects the kidneys and prevents the formation of kidney stones by inhibiting pyroptosis, apoptosis, epithelial–mesenchymal transition (EMT), and macrophage activation. Vitexin also has a protective effect against lipopolysaccharide (LPS)-induced apoptosis of rat renal tubular epithelial cells and reduces cadmium-induced nephrotoxicity. In addition, vitexin inhibited the development of diabetes nephropathy by regulating the NF-κB and AMPK signaling pathways. Data showed that vitexin inhibited ferroptosis and reduced GPX4-related lipid peroxidation by regulating the KEAP1/NRF2/HO-1 pathway, thereby exerting a renoprotective effect [27,29]. In addition, vitexin is considered a candidate drug for cancer treatment because of its ability to inhibit EMT in various cancers. Mechanistic studies have found that vitexin exerts its effects by inhibiting the expression of HMGB1 and activating downstream PI3K/AKT/mTOR/HIF-1 α signaling pathways, providing evidence for potential drugs for the clinical treatment of CKD [21].

APEX1 plays a role in promoting the atheromatous phenotype of the vascular endothelium. The expression of pro-inflammatory cytokines (SELE, VCAM1, and ICAM1) induced by oxidative stress is mediated by APEX1 via the NF-κB pathway. In vivo vitexin administration alleviated neointimal formation and atherosclerosis induced by disturbed blood flow. Through the use of target participation analysis (CETSA) and biophysical properties (SPR), vitexin, as a direct inhibitor of APEX1 in endothelial cells, plays a key role in the anti-inflammatory treatment of atherosclerosis, providing a theoretical basis for the clinical use of vitexin [83].

## 5. Safety Profile and Toxicological Assessment of Vitexin and Isovitexin

Given the potential of vitexin and isovitexin as drug candidates, it is essential to evaluate their safety profiles for human application. At present, a large number of in vitro and in vivo studies have confirmed the safety of vitexin, whereas research on isovitexin is relatively scarce. In vitro experiments have shown that vitexin had no cytotoxicity (IC_50_ > 200 μg/mL). In in vivo studies, high concentrations of vitexin and isovitexin did not show significant toxicity in terms of acute and sub-chronic toxicity, as well as genetic toxicity. Furthermore, in terms of liver and gastric mucosal injury, the long-term repeated use of high-dose vitexin (10 mg/kg, i. p.) was shown to be safe. Vitexin did not cause significant toxic reactions, even at high doses in in vivo experiments [84,85]. These results indicate that vitexin has potential advantages in terms of safety. However, further clinical research on these compounds is necessary to comprehensively evaluate their safety and efficacy, especially for their application in humans.

## 6. Absorption and Metabolism of Vitexin and Isovitexin in Human

Vitexin and isovitexin have poor absorption in the gastrointestinal tract, with significant first-pass effects in the intestine (approximately 94%), stomach (30%), and liver (50%), resulting in a lower bioavailability (F) (approximately 5%). Vitexin and isovitexin directly reach the colon and are hydrolyzed by the gut microbiota through deglycosylation and heterocyclic C-ring opening. The intestinal bacteria *Eubacterium cellulosolvens* can completely convert isovitexin into apigenin by decomposing it into small molecule phenols and various aromatic acids. However, vitexin cannot undergo this complete conversion. This difference may be attributed to the presence of two adjacent hydroxyl groups at the C6 position in isovitexin, which are absent in vitexin [86].

Vitexin is rapidly and widely distributed in various tissues after intravenous and oral administration in rats or mice and has the highest content in the stomach and intestines within 0.5 h, which may be related to residual drugs and enterohepatic circulation. The highest accumulation was observed in the liver. In contrast, the enterohepatic circulation of vitexin also affects its accumulation in the body and it is mainly excreted through the urine. Through intravenous administration in rats, vitexin is mainly distributed in the liver and kidneys, with the lowest concentration in the brain and adipose tissue, and most of it is excreted through the urine and bile. In contrast, after intravenous administration of isovitexin in rats, the levels were highest in the kidneys, liver, and lungs and lowest in the brain. The isovitexin content in the ovary was much higher than that in other organs [86].

The absorption and metabolism processes of vitexin and isovitexin in the human body are complex, and their bioavailability is influenced by multiple factors, including the action of the gut microbiota, interactions with dietary components, first-pass effects, and plasma protein-binding rates. As shown in Table 3, different nano-delivery systems have been developed to enhance the bioavailability of vitexin by improving its solubility, stability, and sustained release properties. The vitexin-loaded bilayer nanoparticles are designed by assembling soybean peptides and coating them with a goblet cell-targeting peptide. They significantly increase the bioaccessibility and bioavailability of vitexin while providing better antioxidant activity through sustained release in the intestine [87]. A *zein*–pectin nanoparticle system exhibits slow-release properties and enhanced absorption in the duodenum, providing a robust delivery system that improves the bioavailability of vitexin, with potential applications in sustained-release formulations [88]. Researchers have also developed D-α-tocopherol polyethylene glycol succinate, polyvinylpyrrolidone K30, and sodium cholate-mixed micelles to enhance the bioavailability and demonstrated the anti-osteoporotic effect of vitexin. The oral bioavailability of vitexin increased by 5.6-fold compared to free vitexin [89]. Another delivery system of mPEG-g-CTS/ALG polyelectrolyte complex nanoparticles enhances vitexin’s gastrointestinal digestion and is suitable for oral, intestinal-specific delivery, offering a new approach for improving vitexin’s absorption and bioavailability [90]. Liposomal encapsulation using the ‘thin-film hydration’ method provides an effective strategy for treating liver cirrhosis by enhancing the bioavailability and therapeutic effectiveness of vitexin through oral delivery [91]. Moreover, the vitexin-rhamnoside (VR) and *zein*-VR-pectin nanoparticles were found to improve the bioavailability of vitexin while alleviating chronic inflammation and hepatic injury in HFD mice [76]. Collectively, these factors determine the distribution and excretion of these two compounds in the body, thereby affecting their efficacy and safety [86,87,88]. Therefore, in clinical applications, it is necessary to optimize administration strategies based on pharmacokinetic characteristics.

## 7. Bioavailability

Vitexin and isovitexin have poor water solubility, resulting in low oral bioavailability and poor gastrointestinal absorption, which limit their effectiveness in vivo. Studies have confirmed that loading it into a nano-delivery system can improve its bioavailability and biological activity. A novel pH-stable targeted oral drug delivery nanosystem was constructed by assembling soy peptides to form a hydrophobic inner layer for loading vitexin and crosslinking the peptide CSKSSDYQC (CSK) targeting goblet cells coupled with N-trimethyl chitosan (TMC) with TPP ions to form an outer layer [87]. By encapsulating vitexin in a corn-*zein*-pectin nanoparticle system, the solubility, stability, and bioavailability of vitexin, as well as the sustained-release properties of the nanoparticles and their high absorption rate in the small intestine, can be improved [88]. D-α-tocopherol polyethylene glycol succinate, polyvinylpyrrolidone K30, and sodium cholate-mixed micelles (Vi MMs) loaded with vitexin improved the oral bioavailability of vitexin and enhanced its anti-osteoporosis effect [89]. Researchers have successfully loaded vitexin into polyethylene glycol methyl ether-grafted chitosan/alginate (mPEG-g-CTS/ALG) nanoparticles, which could enhance their digestion [76,90,91].

The results showed that nanoparticles can protect vitexin from release in the stomach and promote its sustained release in the intestine. In vitro and in vivo experiments showed that nanoparticles significantly improved the bioavailability of vitexin, and vitexin exhibited better antioxidant activity after encapsulation, providing a new strategy for improving the bioavailability of vitexin [76,87,88,89,90,91].

## 8. Prospective

Flavonoids are a class of polyphenols widely present in nature. They have a variety of biological activities, including antioxidant, anti-inflammatory, antitumor, cognitive improvement, cardiovascular protection, and anti-diabetic effects [81,92,93]. These biological activities may be related to the phenolic hydroxyl groups in their molecular structure, especially the adjacent trihydroxy or dihydroxy groups on the A or B ring, which may serve as active functional groups. In addition, the skeletal structure of flavonoids may have good compatibility with certain proteins, although the current evidence is insufficient and requires further validation [1,2,3].

As known flavonoid compounds, the apigenin 8/6-C-glucoside derivatives vitexin and isovitexin not only possess the common biological activities of flavonoids but also exhibit specificity due to their unique chemical structures. For instance, their shared adjacent dihydroxy structure on the A ring facilitates effective free radical scavenging, while the presence of C-8 glucoside enhances the antioxidant capacity of vitexin by reducing the negative charge of the oxygen atom at C-3 [1,2,7]. Recent studies have suggested that these compounds possess a broad spectrum of biological activities, which may contribute to the treatment of cancer, cognitive impairment, depression, Alzheimer’s disease, brain injury, ischemia/reperfusion injury, pain, cardiac hypertrophy, hypertension, diabetes, obesity, infections, and metabolic disorders [81].

These compounds influence numerous physiological processes, including antioxidant, anti-inflammatory [28,29,63], and pro-apoptotic effects [45]. Additionally, they exhibit protective effects on the nervous [33] and cardiovascular systems [4,9,10,20], thyroid gland, liver, and other organs [81] Several biological mechanisms have been proposed to explain their pharmacological actions, such as the apoptotic signaling pathway, inflammatory cytokine network, the MAPK pathway, and key molecular targets including *HIF-1α*, *P53*, and related signaling cascades [28,29,44].

Despite its structural similarity to vitexin, isovitexin has not been extensively studied. Although research on isovitexin has been relatively recent, its potential in antioxidation, anti-inflammation, blood glucose regulation, and liver fibrosis improvement has been demonstrated [81]. As research progresses, isovitexin has emerged as a promising drug candidate for the treatment of various diseases. Further investigations into isovitexin will not only deepen our understanding of flavonoids but also provide essential scientific insights for the development of novel therapeutic agents.

The gut microbiota plays a crucial role in host health by regulating immune responses, preventing pathogen invasion, and influencing the progression of intestinal diseases. Given their anti-inflammatory and antioxidant properties, vitexin and isovitexin may exert beneficial effects by modulating the gut microbiota composition, as shown in Table 4 [15,57]. Future studies should explore the mechanisms through which vitexin and isovitexin interact with the gut microbiota, including their effects on specific microbial populations and their influence on host immune responses and gut barrier function. Additionally, investigating their potential synergy with probiotics and their role in treatment strategies, such as fecal microbiota transplantation, could provide valuable insights.

Thus far, experimental approaches for studying vitexin and isovitexin have included screening assays for medicinal plant extracts, protein-level analyses, observations of cell death and tissue damage, and behavioral tests in animal models. However, most studies have used doses exceeding those typically used for the oral administration of vitexin and isovitexin as plant-derived compounds. Determining the most effective and physiologically relevant dose remains a significant challenge, necessitating further research to facilitate future clinical trials.

## 9. Conclusions

The current understanding of the pharmacological and biological activities, as well as the underlying mechanisms of vitexin and isovitexin, remains limited and warrants systematic investigation. Future research should consider the following:

1. Identification of molecular targets involved in neuroprotection using animal models.
2. Investigation of potential synergistic effects and structure–activity relationships between vitexin, isovitexin, and other therapeutic agents.

Ultimately, in-depth studies encompassing both in vitro and in vivo approaches would enhance our understanding of the pharmacological effects of vitexin and isovitexin. These compounds may serve as promising alternative therapeutic agents for various diseases or as treatments that target specific molecular pathways. Furthermore, vitexin and isovitexin may hold potential as adjunct therapies or preventative health supplements for multiple diseases, paving the way for their broader applications in medicine and healthcare.

## Figures and Tables

**Table 1:** Typical pathways and target cases of vitexin and isovitexin in the treatment of diseases.

Vitexin/ Isovitexin	Biological Activities	Cell/Animal	Dosage	Mechanisms and Pathways	Targets	References
Vitexin	Myocardial ischemia/reperfusion (I/R) injury	Isolated *Sprague-Dawley* rat hearts, H9c2 cells	10 μM	Reducing ROS levels; improving mitochondrial activity, mitochondrial membrane potential, and ATP content; markedly increasing *MFN2* expression and reducing the recruitment of Drp1 in mitochondria.	*MFN2*, Drp1, Epac1-Rap1	[4]
				Inhibiting ischemic myocardial mitochondrial dysfunction and reducing cardiomyocyte apoptosis by regulating Epac1-Rap1 signaling.		[9]
Vitexin	Protect against DOX-induced acute cardiotoxicity	Rats	30 mg/kg	Vitexin induced elevated FOXO3a protein expression levels, by suppressing oxidative stress, inflammation, and apoptotic signals.	FOXO3a	[10]
Vitexin	Pre-eclampsia	Pregnant rats	45–60 mg/kg	Decreased sFlt-1, increased PlGF, and alleviated oxidative stress	HIF-1α, VEGF	[11]
Vitexin/ Isovitexin	Diabetes, overweight	Diabetic rats	1 mg/kg	Inhibits the negative regulator of insulin signaling, protein tyrosine phosphatase (PTP)-1B, inhibits α-amylase and α-glucosidase.	PTP-1B	[12]
Vitexin/ Isovitexin		HepG2				[13]
Vitexin		HUVECs	5–100 μM	Vitexin disrupted Wnt/β-catenin signaling pathway, vitexin activated nuclear factor-erythroid 2-related factor 2 (Nrf2) in HUVEC under high glucose.	Nrf2	[14]
Vitexin/ isovitexin	Diabetes, overweight	HepG2 cells within an insulin-resistant system		Regulating glycemia, through changes in anti-hyperglycemic activity and in the gut microbiota in overweight individuals.		[15]
Vitexin	Diabetic nephropathy	HK-2 cells/DN rat	0–40 μM	Vitexin could alleviate diabetic nephropathy by attenuated ferroptosis via activating GPX4.	GPX4	[16]
Vitexin	Non-alcoholic fatty liver disease	NAFLD mice	6 mg/kg	Vitexin degraded lipids in HFD-induced NAFLD mice liver by inducing autophagy and restoring both ER and mitochondrial biological proteins.		[17]
		CSHFD mice	40 mg/kg	Inhibit TLR4/NF-κB signaling and reduce fatty acid synthesis proteins.		[2]
Vitexin	Obesity	*C57BL/6J*, 3T3-L1 adipocytes	30 mg/kg	Vitexin may prevent HFD-induced obesity/adipogenesis via the AMPKα mediated pathway.	AMPKα	[18]
Isovitexin	Liver fibrosis	Hepatic stellate cell models		Regulation of *miR-21*, targeting PTEN-Akt signaling and the GSH metabolic pathway.	PTEN	[19]
Vitexin	Triple-negative breast cancer			Vitexin promoted M1 polarization and suppressed M2 polarization, affecting EGFR phosphorylation and downstream signaling.	EGFR	[20]
Vitexin	Gastric cancer	Nude mice/GC cells	2 mg/kg 10–160 μM	Vitexin inhibited the malignant progression of GC in vitro and in vivo by suppressing HMGB1-mediated activation of PI3K/Akt/HIF-1α signaling pathway.	HMGB-1	[21]
Vitexin	Colon cancer	HCT-116 cells	1–300 μM	Inhibit colon cancer HCT-116 cell proliferation by suppressing CDK1/cyclin B expression, leading to cell cycle arrest in the G2/M phase.		[22]
Isovitexin	Colon cancer			Promoted apoptosis and suppressed cell proliferation by activating the p53 signaling pathway.		[23]
Vitexin/ isovitexin	Non-small cell lung cancer cells	A549/ H1299 cells, nude mice	1–120 μM	Suppressed NF-κB, AKT and ERK activation.		[24]
Vitexin		A549 cells, nude mice	0–40 μM	Reduced the levels of p-PI3K, p-Akt, and p-mTOR.		[25]
Isovitexin	Hepatocarcinoma	SK-Hep-1 cells		Mediated *miR-34a* upregulation induces apoptosis and suppresses the stemness of SK-SC.	*miR-34a*	[26]
Vitexin	Vitiligo	human melanocyte PIG1	0–40 μM	Protected melanocytes from oxidative stress by activating MAPK-Nrf2/ARE signaling pathway.	Nrf2	[27]
Isovitexin	Acute lung injury	RAW 264.7 cells	0–50 μM	Inhibiting MAPK and NF-κB and activating HO-1/Nrf2 pathways.	Nrf2	[28]
Vitexin	Autoimmune hepatitis	EAH mice	5 mg/kg	Vitexin ameliorated hepatic injury in EAH mice through activation of the AMPK/AKT/GSK-3β pathway and upregulation of the *Nrf2* gene.	*Nrf2*	[29]
Vitexin	Mastitis	MAC-T cells	20 μM	Vitexin inhibited the production of ROS through promoting PPARγ, increased the activity of antioxidant enzymes, and reduced inflammatory cytokines and apoptosis by alleviating ER stress and inactivation MAPKs and NF-κB signaling pathway.	PPARγ	[30]
Isovitexin	Acute gouty arthritis	*Sprague-Dawley* rats	100 mg/kg	Isovitexin ameliorates joint inflammation in acute GA via the TLR4/MyD88/NF-κB pathway.	TLR4	[31]
Isovitexin	Chronic kidney disease	SV40-MES-13 cells/*C57BL/6* mice	0–50 μM, 5 mg/kg	Ameliorated renal injury, inflammation, and increased protected autophagy by anti-ROS production, anti-inflammation, and anti-pyroptosis.		[32]
Vitexin/ isovitexin	Alzheimer’s disease	Microglial cells	0.1–100 μg/mL	Mediating the nuclear factor-kappa B (NF-κB) signaling pathway.	NF-κB	[33]
Vitexin	Cerebral ischemia/reperfusion	Rat	50 mg/kg	Protect the neuron cells and brain related with the Keap1/Nrf2/HO-1 signaling pathway.	Keap1, Nrf2	[34]

**Table 2:** Mechanistic overview of vitexin and isovitexin.

Pharmacological Effect	Compound	Mechanism/Pathway	Target Molecules	Reference
Anti-inflammatory	Vitexin and Isovitexin	Inhibits NF-κB, activates Nrf2/HO-1, AMPK/AKT/GSK-3β	TNF-α, IL-6, IL-1β, Nrf2, ICAM-1, VCAM	[28,29,63]
Antioxidant	Isovitexin	Activates HO-1/Nrf2, reduces ROS, inhibits MAPK	ROS, GPx, SOD, HO-1, CAT	[28,32]
Anti-cancer	Vitexin	Inhibits PI3K/Akt, promotes apoptosis, suppresses HMGB1, modulates Bcl-2/Bax ratio	HMGB1, caspase-3, CDK1, Bcl-2, Bax	[21,22,25]
Hepatoprotective	Vitexin	Modulates Sirt1/p53, reduces apoptosis and lipid accumulation, activates AMPK, enhances IRS-1/AKT signaling	Sirt1, p53, TG, ALT, AST, PPARγ, SREBP-1c	[44,47]
Neuroprotective	Vitexin	Regulates HIF-1-α, MAPK, Keap1/Nrf2, AKT/mTOR, reduces inflammation	VEGF, MMPs, Bcl-2, Bax, caspase-3	[34,71,73]
Cardioprotective	Vitexin	Regulates Epac1-Rap1 pathway, FOXO3a, MAPK/ERK	FOXO3a, *Epac1*, Drp1, *MFN2*	[4,9,10]
Antidiabetic	Vitexin and Isovitexin	Inhibits α-glucosidase/α-amylase, promotes GLUT4, modulates gut microbiota	PTP-1B, GLUT4, GPx4, Nrf2	[12,15,16,40]
Anti-obesity	Vitexin	Activates AMPKα, inhibits C/EBPα, FAS, activates Hedgehog signaling	AMPKα, C/EBPα, FAS	[17,18,41]
Anti-fibrotic	Isovitexin	Suppresses PI3K/Akt, modulates *miR-21* and GSH pathway	*miR-21*, PI3K, GSH, PTEN	[19]
Antimicrobial	Vitexin	Inhibits H+/K+-ATPase, suppresses MPO and biofilm formation, interferes with bacterial efflux pumps	MPO, H+/K+-ATPase	[77,79,80]

**Table 3:** Design types and mechanisms of vitexin-loaded nanocarriers.

Nano Types	Efficacy Tested	Model Type	Results	References
Vitexin-loaded bilayer nanoparticles by the assembly of soybean peptides and the coating of goblet cell targeting peptide CSKSSDYQC (CSK) coupled N-trimethyl chitosan (TMC)	The bilayer nanoparticles could protect vitexin from being released in stomach and promote sustained release in intestine	In vitro	Bioaccessibility and bioavailability of vitexin was significantly increased by the bilayer nanoparticles and vitexin exhibited better antioxidant activity after encapsulation.	[87]
Encapsulated by the *zein*-pectin nanoparticles system	Nanoparticles exhibited significant slow-release properties and the highest absorption rate in the duodenal segment of rats	In vivo/In vitro	It provides a theoretical and technical approach for the construction of vitexin delivery system with sustained-release properties and higher bioavailability	[88]
Vitexin (Vi)-loaded D-ɑ-tocopherol polyethylene glycol succinate, polyvinylpyrrolidone K30, and sodium cholate-mixed micelles	Vi-MMs exhibited enhanced bioavailability and anti-osteoporotic effect	In vivo	The oral bioavailability of Vi-MMs was increased by 5.6-fold compared to free vitexin.	[89]
Vitexin into poly(ethylene glycol) methyl ether-grafted chitosan (mPEG-g-CTS)/alginate (ALG) polyelectrolyte complex nanoparticles.	The gastrointestinal digestion of vitexin increased by encapsulating into mPEG-g-CTS/ALG nanoparticles.	In vitro	Nanoparticles are suitable for oral intestinal-specific delivery systems.	[90]
Vitexin-encapsulated liposomes were synthesized by the ‘thin-film hydration’ method	VLP-treated group also showed better results up to some extent.	In vivo	Liposomal encapsulation of vitexin and subsequent PEG coating to be a substantial strategy for treating liver cirrhosis through oral drug delivery.	[91]
Vitexin-rhamnoside (VR) and *zein*-VR-pectin nanoparticles (VRN)	Alleviating chronic inflammation and hepatic injury in HFD mice.	In vivo	Provided new evidence that nanoparticles enhance the bioavailability of vitexin bioactive ingredients.	[76]

**Table 4:** The regulation and related mechanisms of vitexin and isovitexin on intestinal flora.

Vitexin/ Isovitexin	Disease	Model	Change at Genus Levels	Results	References
Vitexin/ Isovitexin	Overweight	Simulation of Human Gut Model	*Adlercreutzia*, *Terrisporobactel*, *Promicromonospor*, *Pseudonocardia*, *Anaerostipes*, *Akkermansia*, *Alistipes*, *Parabacteroides*, *Enterocloster*, *Peptacetobacter*, *Collinsella*, *Paraclostridium*, *Duncaniella*, *Streptococcus*, *Gillisia*	Industry can use this optimal ratio to formulate more effective functional ingredients for functional foods and create nutraceuticals designed to reduce the risk of T2DM in overweight individuals.	[15]
Vitexin	Acute colitis	An acute colitis mice model	*Veillonella*, *Terrisporobacter*, *Klebsiella*, *Paeniclostridium*, *Parabacteroides*, *Flavonifractor*, *Blautia*	Vitexin could alleviate colitis by regulating gut microbiota and attenuating gut inflammation.	[67]
Vitexin	Neuro-inflammation	Mice model	*Akkermansia*, *Lachnospiraceae*	Vitexin exerted neural protective effects via anti-oxidant, anti-inflammatory, and gut microbiota modulating properties.	[57]
Vitexin	Lipid metabolism disorders		*Rombousia* and *Faecalibaculum*	Vitexin can regulate the gut microbiota and thus improve lipid metabolism.	[76]
